# The Genomic Basis of the Svalbard Reindeer's Adaptation to an Extreme Arctic Environment

**DOI:** 10.1093/gbe/evaf160

**Published:** 2025-08-13

**Authors:** Nicolas Dussex, Vanessa C Bieker, Xin Sun, Mathilde Le Moullec, Erik Ersmark, Knut H Røed, John R Speakman, Leif Egil Loe, Love Dalén, Brage B Hansen, Michael D Martin

**Affiliations:** Department of Natural History, NTNU University Museum, Trondheim 7012, Norway; Department of Population Analysis and Monitoring, Swedish Museum of Natural History, Stockholm 114 18, Sweden; Department of Natural History, NTNU University Museum, Trondheim 7012, Norway; Department of Natural History, NTNU University Museum, Trondheim 7012, Norway; Center for Evolutionary Hologenomics, The GLOBE Institute, University of Copenhagen, København 1350, Denmark; Gjærevoll Centre for Biodiversity Foresight Analyses, Norwegian University of Science and Technology (NTNU), Trondheim 7034, Norway; Department of Mammals and Birds, Greenland, Institute of Natural Resources, Nuuk 3900, Greenland; Centre for Palaeogenetics, Stockholm SE-106 91, Sweden; Department of Bioinformatics and Genetics, Swedish Museum of Natural History, Stockholm 114 18, Sweden; Department of Zoology, Stockholm University, Stockholm 114 18, Sweden; Department of Preclinical Sciences and Pathology, Norwegian University of Life Sciences, P.O. Box 5003 Ås 1432, Norway; School of Biological Sciences, University of Aberdeen, Aberdeen AB24 2TZ, UK; Faculty of Environmental Sciences and Natural Resource Management, Department of Ecology and Natural Resource Management, Norwegian University of Life Sciences (NMBU), P.O. Box 5003, 1432, Ås, Norway; Centre for Palaeogenetics, Stockholm SE-106 91, Sweden; Department of Bioinformatics and Genetics, Swedish Museum of Natural History, Stockholm 114 18, Sweden; Department of Zoology, Stockholm University, Stockholm 114 18, Sweden; Gjærevoll Centre for Biodiversity Foresight Analyses, Norwegian University of Science and Technology (NTNU), Trondheim 7034, Norway; Department of Terrestrial Ecology, Norwegian Institute for Nature Research (NINA), Trondheim 7034, Norway; Department of Natural History, NTNU University Museum, Trondheim 7012, Norway

**Keywords:** adaptation, genomic, Arctic, climate

## Abstract

Studying adaptation to extreme climates is essential for understanding evolutionary processes and how species evolve and persist under changing environmental conditions, such as climate warming. Here, we investigate the genomic basis of adaptations in the Svalbard reindeer (*Rangifer tarandus platyrhynchus*), an endemic subspecies that colonized the High Arctic approximately 7,000 years ago and developed a suite of adaptations for survival under conditions of extreme cold, changes in day length, and resource scarcity. Applying scans of selection, functional analysis of coding region variation, and characterization of copy number variation across reindeer populations from Svalbard, mainland Norway, mainland Russia, and Novaya Zemlya, our comparative genomics approach identified 150 genomic regions that are differentiated in Svalbard reindeer relative to mainland reindeer (*R. tarandus*). These genomic regions include genes linked to fat metabolism, energy conservation, cold tolerance, body size, fur morphology, and seasonal circadian rhythm. Our study highlights the advantages of using distinct approaches to uncover the genomic basis of adaptations and provides a path for future research into the evolution of species in similar environments.

Significance Identifying adaptations to extreme and novel environments is crucial to understand how species can adapt to rapid environmental changes. Our analysis of Svalbard reindeer genomes and those from other mainland reindeer successfully identified several genes associated with fat metabolism, energy conservation, cold tolerance, body size, fur morphology, and seasonal circadian rhythm that presumably appeared over the past 7,000 years, since the colonization of the archipelago. Our findings provide important insights into the molecular evolution involved in phenotypic changes that potentially contribute to the adaptation to extreme climates over relatively short evolutionary time scales.

## Introduction

How small populations adapt to novel environments is a central question in evolutionary biology. Due to their geographical isolation, historical founder effects, and differences in environmental conditions compared to their original range, island populations provide unique opportunities to study the evolution of small populations (Losos and Ricklefs 2009).

The colonization of island ecosystems is often associated with remarkable phenotypic adaptations associated with different selective pressures in the novel environments. For instance, the island rule (ie Foster's rule) posits that dwarfism or gigantism is a common feature in island vertebrates (Lomolino 1985, 2005). The main mechanisms invoked to explain this rule are a low number of predators and relaxed interspecific competition (ie ecological release) or, in contrast, limited resources (Lomolino et al. 2012). While ecological release favors gigantism in small-bodied species, certain climatic conditions and resource limitations select for smaller body sizes with reduced energy requirements, leading to insular dwarfism, as shown in a number of mammalian species (Lomolino 1985; Benítez-López et al. 2021). Furthermore, according to Allen's rule, endothermic animals from colder climates have a lower surface area-to-volume ratio and thus shorter appendages (ie ears, tails, and limbs) relative to species from warmer climates (Allen 1877). This adaptation helps regulate body temperature by minimizing heat loss in cold environments or by maximizing heat dissipation in warmer ones. Distinct climates and varied resources will also select for adaptations allowing the exploitation of a new available niche (Losos and Ricklefs 2009). For instance, harsher climatic conditions and limited resources will select for the ability to exploit new and less varied food sources as well as favor more efficient metabolism or thermoregulation functions (Blix 2016). Importantly, colonization of new environments often involves lone or few dispersal events and individual founders, which will result in a reduction in genome-wide diversity through the effects of genetic drift (Mayr 1954; Nei, Maruyama, and Chakraborty 1975). Yet, striking phenotypic adaptations can still evolve from low-standing genetic variation associated with a small number of founders (Grant 1998).

As a case in point, the Svalbard reindeer (*Rangifer tarandus platyrhynchus*) colonized the Svalbard archipelago in the Holocene and has developed a suite of remarkable adaptations to the High Arctic (Staaland et al 1988; Hansen and Aanes 2015). Ancient reindeer feces and antlers as well as genomic data suggest that reindeer colonized the archipelago at least 7,000 years before present (van der Knaap 1989; Sahlman et al. 2009; Dussex et al. 2023; Hold et al. 2024). Moreover, whole-genome data indicate that this colonization was probably the result of a small founder population comprising fewer than 100 individuals (Dussex et al. 2023) that arrived in the archipelago from Russia via Franz Josef Land (Kvie et al. 2016; Dussex et al. 2023; Hold et al. 2024). Since then, the Svalbard population has been subject to long-term isolation and inbreeding, and a near-extinction event due to overharvest in the 20th century (Hansen and Aanes 2015; Kellner et al. 2024). These processes facilitated the purging of some of its genetic load and contributed to a severe loss of genetic diversity relative to its ancestral mainland population (Dussex et al. 2023).

**Fig. 1.**
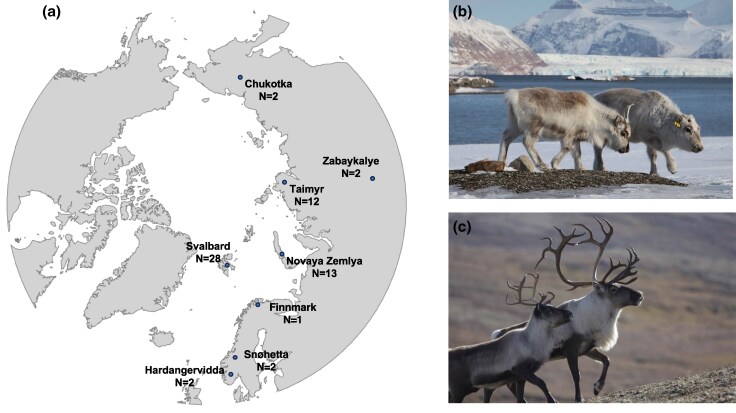
Sample distribution and phenotypes of *R. tarandus*. a) Sample origin of the 62 genomes analyzed. Photographs display examples of the distinct phenotypes of b) Svalbard reindeer (*R. t. platyrhynchus*) relative to c) Eurasian reindeer (*R. t. tarandus*). Photo credits: Brage B. Hansen (b) and Roy Andersen (c).

In spite of being derived from a small founder population and having low genetic diversity, the Svalbard reindeer has adapted to its extreme arctic environment over a relatively short evolutionary time period. For instance, it has reduced head and body size, shortened extremities (ie tail and ears) and legs, thick fat layers, and a highly insulating fur, all of which are believed to grant the reindeer a special tolerance to cold and wind (Cuyler and Øritsland 2002b; Fig. 1). The Svalbard reindeer also experiences seasonal changes in foraging behavior, metabolic activity, thermoregulation (Trondrud et al. 2021a, 2021b), and circadian rhythmicity that can be viewed as adaptations to the highly seasonal conditions in the High Arctic (Arnold et al. 2018). Furthermore, in contrast to its mainland counterparts (ie *R. t. tarandus*) that rely on lichens as their main winter food source and since lichens are almost totally absent from the archipelago, the Svalbard reindeer's diet comprises a large proportion of bryophytes and vascular plants (Staaland et al. 1988). Finally, the subspecies is not gregarious and does not undertake migrations, but instead they are relatively tame, solitary, and stationary, consistent with their evolution more or less in the absence of predators (Hansen and Aanes 2015). However, we lack knowledge on the genomic basis of these adaptations.

Here, we use a comparative genomics approach and analyze complete reindeer genomes from Svalbard, mainland Norway, mainland Russia, and Novaya Zemlya to examine the genomic basis of adaptation in this insular subspecies using three distinct approaches. We hypothesized that changes in multiple genetic pathways have contributed to the adaptation process, potentially including genes involved with body and bone development, energy metabolism and fat storage, nutrient digestion, and behavior.

## Results and Discussion

### Population Structure and Phylogeny

We sequenced 62 genomes of wild *R. tarandus* individuals from Svalbard, mainland Norway, mainland Russia, and Novaya Zemlya (Table S1) with a depth of coverage ranging from 4 to 35× (median = 11) and varying across sampling localities.

**Fig. 2.**
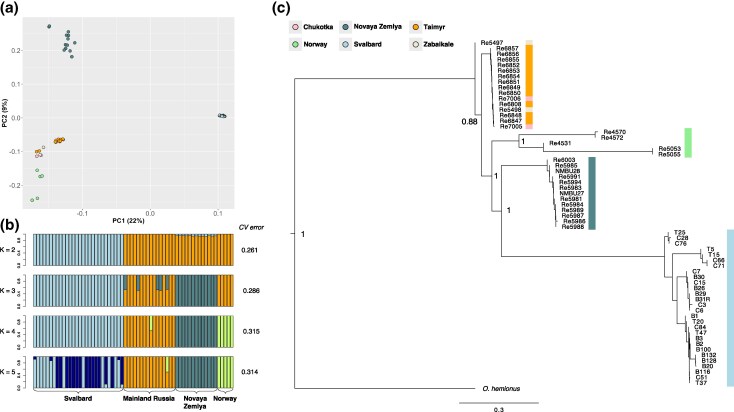
Population structure for the 62 reindeer genomes analyzed. a) Principal component analysis (PCA). b) Admixture plots for *K* = 2 to 5. c) Neighbor-joining phylogeny using mule deer (*Odocoileus hemionus*) as outgroup with bootstrap values obtained from 100 bootstrap replicates.

Principal component analysis (PCA) and admixture analysis based on 19,829,994 single-nucleotide variants revealed three main clusters: Svalbard, Novaya Zemlya, and Eurasia (ie mainland Norway and Russia; Fig. 2a and b). The neighbor-joining and maximum-likelihood phylogenies (Fig. S1) confirmed that Svalbard reindeer represents a monophyletic clade, with Novaya Zemlya, mainland Russia, and mainland Norway representing sister clades (Fig. 2c). This phylogeny is consistent with a wider Eurasian phylogeny based on mitochondrial DNA (Hold et al. 2024), whereas the admixture analysis (Fig. 2b) indicates that Novaya Zemlya reindeer are not admixed and thus represent an appropriate group for our tests of selection.

### Putative Genes Underlying the Svalbard Phenotype

**Fig. 3.**
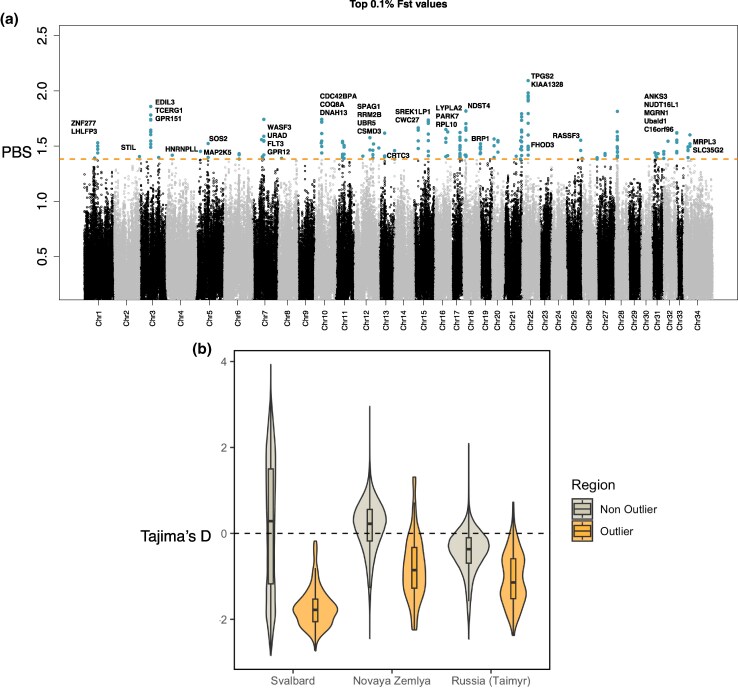
Genome scans of selection using the population branch statistic (PBS) and neutrality tests. a) Blue dots represent values above the 99.9th percentile shown with a horizontal dashed orange line (Table S2). Names of genes within the highest peaks are shown. b) Neutrality statistics with Tajima's D for outliers and nonoutlier regions from (a).

We used three approaches to identify coding regions that may underlie the unique phenotypes of Svalbard reindeer. First, we used the population branch statistic (PBS), which uses pairwise *F*_ST_ values among a set of three populations with known phylogenetic relationship to detect genomic regions under putative positive selection in a focal population. With Svalbard as the focal population, we compared the allele frequency differences between Svalbard reindeer genomes (*n* = 28) and Russian genomes (Novaya Zemlya, *n* = 13; Taimyr, *n* = 12). Retaining the top 0.1% PBS values, we found 75 outliers overlapping with coding regions (Fig. 3a; Table S2). To differentiate between outlier regions resulting from positive selection or genetic drift associated with the recent bottleneck, we calculated Tajima's D (Tajima 1989). For non-outlier regions, Svalbard and Novaya Zemlya showed the highest proportion of windows with positive values, and mean values slightly above zero, consistent with histories of population bottlenecks and founder effects when colonizing their respective islands (Fig. 3b). In contrast, outlier windows showed primarily or entirely negative values for all three populations, with the lowest mean value in the Svalbard population, which is consistent with purifying selection or selective sweeps.

Secondly, we sought to identify candidate genes that encode functionally relevant derived variants that have risen to high frequency in the Svalbard population. To find these variants, we used SNPeff to annotate nonsynonymous variants within the coding regions of all 31,659 genes and retained SNPs as candidates under putative recent positive selection if the frequency of derived alleles was ≥0.7 in Svalbard genomes and ≤0.25 in mainland Russian (ie Taimyr) and Novaya Zemlya genomes, and to take into account the effects of drift associated with the recent population bottleneck and colonization of Svalbard (see Materials and Methods). We identified one variant predicted to have high impact (ie lost stop codon) and 54 variants predicted to have moderate impact (ie missense; nondisruptive variants that might change protein effectiveness) within 44 genes (Table S3). However, it should be noted that using low-coverage data (ie 4 to 5×) for the SNPeff analysis may increase the rate of genotyping errors and thus also the rate of false-positive amino acid substitutions. In order to mitigate this, we used several individuals per population, only used high-quality base calls, and applied strict variant filtering, thereby excluding low-confidence regions. In contrast, the risk of false negatives due to missed heterozygous sites, stochastic allele sampling, and insufficient read support is higher with low-coverage data, even with stringent filtering. Consequently, we cannot exclude the possibility that there may be undetected variants under putative selection, especially if they are rare variants.

**Fig. 4.**
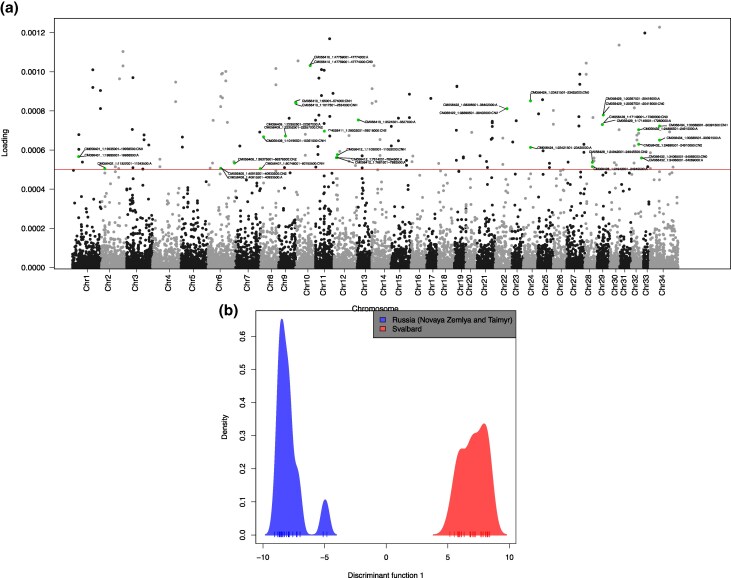
Discriminant analysis of principal components (DAPC) based on copy number variants (CNVs). a) Loadings of each CNV allele in the DAPC analysis. The horizontal red line indicates the threshold for defining outlier CNVs. Green dots with tags indicate outlier CNVs overlapping with genes and are annotated with the CNV ID and allele with CN0 indicating a deletion, CN2 a duplication, CNH more than two copies, and A the ancestral (single-copy) allele (Table S4). The start position of a CNV was used for plotting. b) Density distribution of the Russian (blue) and Svalbard (red) population over the first DAPC axis.

Third, we performed an analysis of structural variation based on depth of coverage, yielding a list of copy number variants (CNVs) and large deletions detected in the panel of genomes. Out of a total of 11,525 CNVs identified in our dataset, we found 124 duplications and deletions (ranging in length from 2 to 113 kbp) that were highly differentiated between Svalbard and Russia (ie mainland/Taimyr and Novaya Zemlya) based on discriminant analysis of principal components (DAPC) (Fig. 4). These included four deletions and 10 duplications overlapping identifiable genes and at high frequency in Svalbard reindeer (Table S4). We also identified large deletions (>500 bp) fixed in Svalbard reindeer relative to Russian and Norwegian reindeer overlapping with two genes (Table S5). Finally, we note that among the results of our three approaches, we did not find any overlapping coding regions under putative selection (Fig. S2), suggesting that the genomic architecture of adaptive variation may vary among functions and genes and also among reindeer subspecies. Recent studies in oak trees and ruminants found little overlap between candidate SNPs and structural variants (SVs) putatively under selection, suggesting that SNPs and SVs may be independently selected (Cumer et al. 2021; Liang et al. 2025). Our results thus suggest that this may also be the case for SNPs and CNVs in reindeer.

### Gene Ontology

**Fig. 5.**
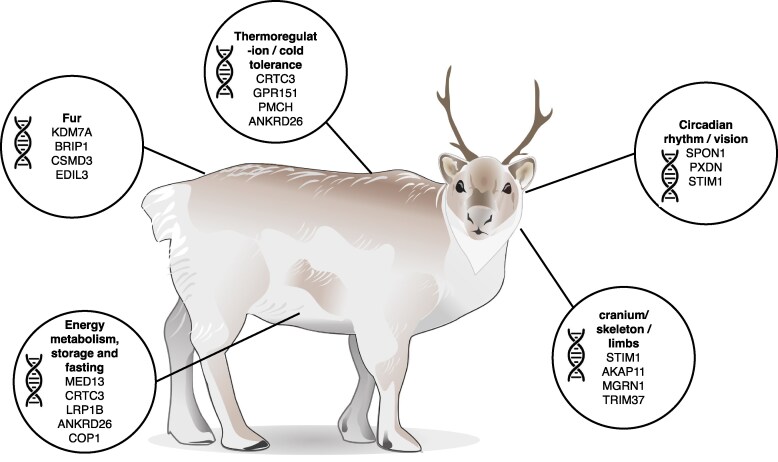
Depiction of the main biological functions identified using comparative genomics, genome scans, and structural variation analysis (Table S7).

Our three approaches to identify regions underpinning the phenotype of Svalbard reindeer yielded a combined list of 150 regions under putative selection, with 120 corresponding to genes with an identifier (Table S2 to S5). A gene ontology enrichment analysis for those 120 candidate genes identified eight statistically overrepresented biological functions (Mi et al. 2010) such as methylglyoxal metabolism (associated with insulin secretion and resistance), regulation of DNA repair, transport, metabolism, and microtubule extension (Table S6). Moreover, we note that there is no obvious overrepresentation of any function in the candidate selected genes identified by any specific method, although there were only a few functions identified in the CNV analysis, owing to the smaller number of candidate genes discovered by this method. Examination of individual candidate genes under putative selection using literature searches and relevant mammalian phenotypes in the Mouse Genome Informatics (Blake et al. 2014) database revealed several biological functions potentially associated with adaptation to the High Arctic environment and including energy metabolism, thermoregulation, and morphology (Blix 2016) (Fig. 5; Table S7).

#### Energy Metabolism and Storage and Fasting

Fat storage is an essential function in arctic environments in both birds and mammals (Blix 2016). Fat content is kept in store during periods of low resources and fasting and shows a seasonal decrease as winter progresses, as observed in both Norwegian mainland and Svalbard reindeer (Larsen et al. 1985; Tyler 1987). Survival is contingent on making energy reserves last until new fresh plant growth is available (Trondrud et al. 2021b). Additionally, there is a close association between fat reserves and pregnancy in reindeer, caribou (Thomas 1982), and muskox (Adamczewski et al. 1998), suggesting that adaptation to conserve energy is a key element for reproductive success (Tyler 1987; Albon et al. 2017).

Our analysis of genomic variants identified several genes associated with energy storage, fat deposition, body mass index (BMI), and obesity in humans with fixed or high-frequency missense mutations (eg BCO2, Våge and Boman 2010; GPTA2, Mitka et al. 2019; LRP1B, Lee 2019; AKAP9, Blake et al. 2014; Table S7), high allelic differentiation based on the PBS (eg IPO8, Hurtado del Pozo et al. 2010; MED13, Amoasii et al. 2016; CRTC3, Liu et al. 2021, 2018) or evidence for CNV (eg ANKRD26, Bera et al. 2008; Acs et al. 2015; Raciti et al. 2017) unique to the Svalbard reindeer population. For instance, MED13 and CRTC3 enhance muscle glucose uptake and storage as glycogen (Amoasii et al. 2016; Liu et al. 2018), and the increase or decrease in expression of MED13 can cause leanness or obesity (Lee et al. 2014). Importantly, a number of those genes (ie CRTC3, MED13, PMCH, and ANKRD26) are involved in metabolism of leptin, a hormone responsible for both body fat and energy conservation in several vertebrate species (Blix 2016). For instance, feeding experiments on reindeer show that leptin levels (as well as insulin) are reduced during periods of undernutrition and starvation and are also controlled by photoperiod length (Soppela et al. 2008).

Consistent with the prolonged scarcity of winter food resources on Svalbard, we also identified genes directly associated with fasting (eg MED13, Amoasii et al. 2016; Lee et al. 2014; TRIM37; WDR36; Table S7). For instance, transgenic MED13 mice adapt to fasting by being more efficient at oxidizing fat fuel reserves (Baskin et al. 2014). Interestingly, prolonged periods of food deprivation can also lead to intermittent insulin resistance and thus spare glucose, as shown in fasting northern elephant seals (*Mirounga angustirostris*; Viscarra et al. 2011; Martinez and Ortiz 2017). It is thus not surprising that we also identified a number of genes involved with insulin metabolism (eg LRP1B, Burgdorf et al. 2012; TCERG1, Nakano et al. 2020; MGRN1, Phan et al. 2006; ANKRD26, Raciti et al. 2011). For instance, LRP1B and ANKRD26 are associated with insulin resistance in mice and humans (Bera et al. 2008; Burgdorf et al. 2012). It has been hypothesized that insulin resistance in peripheral organs may have evolved as a physiological adaptive mechanism in which adipose tissue-derived fatty acids are used to cover the energy needs of peripheral organs, whereas glucose is spared for the brain (Tsatsoulis et al. 2013). Moreover, we found a deletion in one gene (constitutive photomorphogenesis protein 1, COP1; Table S4) which regulates fasting metabolism and feeding-to-fasting transition (Sanchez-Barcelo et al. 2017). During feeding, COP1 expression will inhibit gluconeogenesis and lipolysis, whereas during fasting, a reduction in COP1 expression will increase the rate of these processes. Thus, a deletion in this gene could alter lipid metabolism and insulin signaling during fasting. However, given that the absence of COP1 is lethal to mouse embryos (Migliorini et al. 2011), it is unclear how the loss of this gene is tolerated in Svalbard reindeer, and knockout experiments would be required to fully assess the impact of such deletion on metabolism.

#### Thermoregulation and Cold Tolerance

In the cold climate of the High Arctic, the maintenance of body temperature is crucial. Svalbard reindeer are known to modulate their seasonal energy requirements such as heat production by reducing their heart rate and heat loss in winter (Trondrud et al. 2021a, 2021b). Fat deposition is also important for thermoregulation as fat is used both as thermal insulation and, in the case of brown adipose tissue, can generate heat (Ishida et al. 2024). Examples of convergent evolution in arctic ruminants (ie reindeer and muskox) have been identified for genes associated with brown adipose tissue thermogenesis and fat metabolism (Li et al. 2023). Here, we found a number of genes putatively under selection in Svalbard reindeer related to fat metabolism. For instance, a knockout mutation in CRTC3 results in a reduction of brown adipose tissue activity in mice by downregulating sympathetic nerve activity and vascularization in this tissue, thus making them more cold-tolerant (Yoon et al. 2018). Furthermore, the gene GPR151, identified in the PBS analysis, is associated with neuronal function and pain tolerance (Xia et al. 2021). In mice, a knockout mutation in this gene causes a reduction in cold- and warm-induced pain relative to wild-type mice, suggesting that when functional, this gene plays an important role in cold sensitivity. This phenotype may be an example of convergent evolution (ie the evolution of a similar phenotype involving different genes) with the woolly rhinoceros, another cold-adapted species in which a mutation in the KCNK17 gene allows the positive regulation and expression of the temperature perception gene TRPA1 (Lord et al. 2020).

While the hormone leptin (ie CRTC3, MED13, PMCH, and ANKRD26) is associated with fat metabolism and energy conservation, it also plays an important role in regulation of body temperature in humans, with a positive correlation between leptin levels and maintenance of body temperature (Nikanorova et al. 2020). Moreover, humans from temperate regions have higher levels of leptin compared to their subtropical counterparts (Nikanorova et al. 2020 ), whereas leptin deficiency is associated with mild hypothermia (Farooqi et al. 1999).

Finally, consistent with the Svalbard reindeer's specific fur coat adaptations that favor insulation (Fig. 1), including seasonal changes in fur length and depth (Cuyler and Øritsland 2002a), we found several candidate genes under putative selection associated with fur and coat/skin function and development (eg KDM7A, BRIP1, CSMD3, and EDIL3). For instance, KDM7A is associated with abnormal hair follicle morphology in mice (Liakath-Ali et al. 2014).

#### Morphology and Dwarfism

A characteristic feature of Svalbard reindeer is its reduced nose and body size (Hakala et al. 1986), which is predicted to reduce heat loss in accordance with Allen's rule (Allen 1877). Moreover, shorter legs can reduce heat loss by making the countercurrent vascular heat exchange more efficient (Blix 2016) and may also represent an adaptation to open terrain (Pelletier et al. 2021). We found a number of genes associated with bone density and development or cartilage (eg AKAP11, DCHS1, FLT3, SPON1, STIM1, and EDIL3). For instance, STIM1 and AKAP11 are associated with tibia diameter and length as well as body size and bone mass (Correa-Rodríguez et al. 2018; Silva-Rojas et al. 2019; Table S7). Additionally, mutant mice for the UBR5 gene have shorter limbs, suggesting that this gene is involved in embryonic limb development (Kinsella et al. 2016). Interestingly, we found one gene associated with nanism/dwarfism in humans (TRIM37, Hämäläinen et al. 2006) as well as two genes associated with a small-cranium phenotype (MGRN1, TRIM37; Blake et al. 2014).

#### Other Functions

One key feature of the Svalbard environment is the extremely reduced day length during winter, with ca. 100 to 150 d per year during which the sun does not rise above the horizon. Consistent with this constraint in day length, a previous study identified a number of genes associated with circadian rhythm regulation under selection in reindeer and muskox, although signature of selections did not affect the same genes in these two species (Li et al. 2023). Here, we identified a missense mutation in the gene SPON1, whose protein product is associated with maintaining intrinsic circadian rhythm (Carrillo et al. 2018). For instance, F-spondin-deficient mice lose their ability to maintain typical intrinsic rhythmicity. This variant is fixed in the Svalbard subspecies and entirely absent in the Russian population. Furthermore, we found one allele at high frequency in Svalbard reindeer in the PXDN gene (Yan et al. 2014; Kim et al. 2019) and one outlier from the PBS analysis overlapping with the STIM1 gene (Silva-Rojas et al. 2019), which are both associated with eye or optic nerve development. Arctic reindeer (*R. t. tarandus*) eyes are able to adapt to winter light conditions, extending the visual range into the shorter end of the spectrum to utilize the UV light available during periods of extended twilight characteristic for spring and autumn at high latitudes (Hogg et al. 2011; Fosbury and Jeffery 2022). Consequently, adaptation to low-light conditions may have evolved several times in *R. tarandus* and could be underpinned by several genes, with some mutations able to increase the plasticity of genes associated with vision.

Finally, we found putative genes under selection underpinning biological functions including reproduction and fertility (see piRNA biosynthetic process; Table S6), embryogenesis and development, cardiac and vascular system (Table S7).

## Conclusion

Using a combination of several approaches, we identified several key mutations underpinning adaptations of Svalbard reindeer to the arctic environment. We show that biological functions related to fat and energy metabolism, cold tolerance and thermoregulation, and smaller body size are under putative selection. Furthermore, our results highlight the advantage of using several approaches for the identification of biological functions under selection.

However, among these approaches, we did not detect any overlapping genes under selection, which could be due to fundamental differences in the signals that each approach can detect. Genome-scanning methods like PBS are highly sensitive to population differentiation, often capturing signals across the whole genome, including noncoding or regulatory regions, but at the cost of a higher false-positive rate (Lotterhos and Whitlock 2015; Hoban et al. 2016; Wellenreuther and Hansson 2016). In contrast, approaches such as SnpEff and CNV identification methods focus on functional or structural impacts with greater specificity, reducing false positives but potentially missing genuine signals of adaptation that lie outside annotated coding regions or fall below detection thresholds in low-coverage data (Lotterhos and Whitlock 2015; Hoban et al. 2016; Wellenreuther and Hansson 2016).

Nevertheless, several important questions remain unanswered. First, another reindeer subspecies has adapted to other islands of the High Arctic (eg Peary caribou, *R. t. pearyi*) and also has a reduced head and body size relative to their mainland counterpart. A comparative genomic approach could thus allow testing for convergent evolution and may be better able to identify the environmental and ecological factors driving selection toward such phenotypes. Second, the investigation of gene expression (ie RNA sequencing) and regulatory regions (ie upstream and downstream variants) could reveal additional functions under selection and reveal in detail the tissues where the selected genes play a role in the adaptation to the environment of the Svalbard archipelago. Furthermore, while the PBS approach is powerful in its ability to detect genomic regions under selection (Yi et al. 2010; Shpak et al. 2025), there are still uncertainties about the proportion of unique variation which is the result of positive selection rather than genetic drift, owing to the long history of small population size in Svalbard (Dussex et al. 2023). For instance, a previous study using historical and ancient genomes of Svalbard reindeer identified a number of outlier genomic regions associated with lipid metabolism, spermatogenesis, and circadian rhythm. However, these most likely evolved under genetic drift, and those authors concluded that positive selection had been stronger up until the start of intensive human hunting of reindeer (Kellner et al. 2024 ). Future studies using temporally spaced ancient genomes from 7,000 years BP onward could provide a better understanding of the ancestral state of the Svalbard reindeer genome, produce a timeline for the appearance of the specific adaptations found in modern populations, and help to differentiate how the variants we identify evolved under genetic drift or natural selection or some combination of the two processes. Future work could also determine whether the functional variation in the candidate genes associated with adaptations to the Svalbard environment is sufficient to withstand demographic fluctuations and environmental changes in the face of current climate change, through the use of ancient DNA (Dehasque et al. 2020) and simulations (Haller and Messer 2023). We also note that generating long-read sequencing data would provide valuable validation of the copy number variants identified in this study.

Finally, while we identified a number of candidate genes potentially underpinning important adaptations in Svalbard reindeer, it is important to note that those gene functions were originally inferred in experimental work (eg knockout studies) on mice or other mammals or from gene ontology analyses. There is thus some degree of uncertainty on whether the reindeer variants actually have a similar effect on phenotype in Svalbard reindeer or whether they might affect the expression of other genes with more distinct functions, which further highlights the need for future work on gene expression. Nevertheless, due to the high degree of gene conservation among mammals (Rosen et al. 2015), we have grounds to expect their phenotypic effects are reasonably similar in Svalbard reindeer, and the exact biological functions of the identified candidate genes and variants can be pursued in future experimental studies (eg knockout experiments in mice).

## Materials and Methods

### Sampling

Some Illumina platform genomic data utilized in the study were sourced from the European Nucleotide Archive (PRJEB57293, PRJEB61721, and PRJEB37216; Table S1). Including new sequencing data reported in this study (PRJEB80888), our dataset comprised *R. t. platyrhynchus* genomes from Svalbard (*n* = 28) and *R. t. tarandus* genomes from Siberian Russia (*n* = 16), Novaya Zemlya (*n* = 13), and mainland Norway (*n* = 5; Table S1).

### DNA Extraction and Sequencing

For samples newly sequenced for this study, genomic DNA was extracted from 20 mg muscle tissue using a DNeasy Blood & Tissue kit (Qiagen, Hilden, Germany). Genomic libraries for the Novaya Zemlya and Taimyr samples were prepared by Novogene UK following random shearing and then sequenced on the Illumina NovaSeq 6000 platform using 2× 150 bp chemistry at Novogene UK. For the single sample from mainland Norway, DNA was randomly sheared into short fragments of mean length 400 bp using a Covaris ME220 focused ultrasonicator, and a genomic library was prepared in-house using a blunt-end protocol for double-stranded DNA (BEST v1.1; Carøe et al. 2018) as described in Burnett et al. (2023). This library was sequenced on an Illumina HiSeq 4000 using 2× 150 bp chemistry at the NTNU Genomics Core Facility in Trondheim, Norway.

### Genomic Data Mapping

Data mapping to a chromosome-level caribou (*R. t. caribou*) assembly (GCA_019903745.2) and variant calling genomes was done using the GenErode pipeline (Kutschera et al. 2022). Adapter trimming was done with fastp v0.22.0 (Chen et al. 2018), read mapping with BWA v0.7.17 (Li and Durbin 2010) *mem*, and PCR duplicates removal with SAMtools v1.12 (Li et al. 2009). Reads were realigned around indels using GATK *IndelRealigner* v3.4.0 (McKenna et al. 2010).

We used bcftools v1.8 (Li et al. 2009; Li 2011) *mpileup* to call variants, which were filtered using a minimum depth of coverage (DP4) of ∼⅓ (ie 5×) of the average depth of coverage, and base quality QV ≥ 30. SNPs within 5 bp of indels were removed. To avoid biases caused by contamination, mapping or sequencing error, we filtered out SNPs in a heterozygous state that were not in an allelic balance (ie number of reads displaying the reference allele/depth) of <0.2 and >0.8. After merging all individual vcf files, we excluded the X chromosome (GCA_019903745.2-CM056435.1) and obtained 19,829,994 variant sites.

### Gene Annotations

We first retrieved gene models from the Svalbard reindeer annotation (ie GCA_949782905.1; 32,656 gene models) with gffread from the Cufflinks v2.2.1 (Trapnell et al. 2012) package. We then used spaln2 v2.3.1 (Iwata and Gotoh 2012) to annotate gene models with the following parameters: -Q7 -LS -O0 -S3 -M1 -pF, obtaining a total of 31,659 genes.

### Population Structure and Phylogeny

We first performed a principal component analysis (PCA) in PLINK v2 (Chang et al. 2015) using the hard call genotypes from GenErode. We also estimated individual-based ancestry to infer the number of distinct genetic clusters with ADMIXTURE v1.3.0 (Alexander et al. 2009) for *K* = 2 to 5 and using the cross-validation error estimation (--cv option). Next, we built a neighbor-joining phylogeny with PCAngsd (Meisner and Albrechtsen 2018) using the covariance matrix estimated based on individual allele frequencies. Genotype likelihoods for each genome in ANGSD (Korneliussen et al. 2014) including for mule deer (*Odocoileus hemionus*) used as outgroup, were estimated using the following options: *-GL 2 -doGlf 2 -doPlink 2 -doMajorMinor 1 -SNP_pval 1e-6 -doGeno −1 -doPost 1 -minMapQ 30 -minQ 20 -minMaf 0.05 -doCounts 1 -doMaf 3 -geno_minDepth 5 -setMinDepthInd 5 -geno_maxDepth 8 -postCutoff 0.95 -minInd 30 -remove_bads 1 -uniqueOnly 1*. To estimate bootstrap values, 100 bootstrapped Beagle files were generated using the sample() function in Python with replacement. These Beagle files were then used in a PCAngsd run as described above. The resulting trees were used in R v4.3.1 (R Core Team 2020) to add bootstrap values to the tree using the *prop.clades()* function from the ape package.

Secondly, we generated a nuclear maximum-likelihood (ML) phylogeny of the nuclear genome using *O. hemionus* as an outgroup. We filtered out linked SNPs from the vcf file using plink v2.00a3.7 with the --indep-pairwise option using a window size of 50 bp, a step size of 3 bp, and a threshold of 0.3 and keeping sites with a maximum missingness across samples of 0.2 (*--max-missing* 0.8) using VCFtools v0.1.16 (Danecek et al. 2011). We extracted the chromosomal region in fasta format using SAMtools v.1.18 *faidx* and BCFtools v1.18 *consensus* with missing genotypes set to “N.” Sites not present in the vcf file were removed from the resulting fasta files which were then combined into a single fasta alignment per chromosome. Chromosomal alignments were then concatenated using Geneious Prime v2023.0.1 (https://www.geneious.com). The final alignment contained 32,130 sites. A maximum-likelihood phylogeny was generated with raxml-ng v. 1.1.0 with the GTGTR + G model using 20 random and 20 parsimonious starting trees with 100 bootstraps. The resulting phylogeny was plotted in R v4.3.1 (R Core Team 2020) using the ggtree package.

### Genome Scans of Selection and Neutrality Tests

We used the population branch statistic (PBS; Yi et al. 2010) to detect signals of positive selection in Svalbard reindeer. PBS is based on the lineage-specific *F*_ST_ and compares allele frequency changes between two sister populations and an outgroup population to identify genomic regions under recent natural selection and has been shown to be powerful at identifying outliers under putative selection in the presence of drift (Shpak et al. 2025). We compared the Svalbard reindeer (*n* = 28) to Novaya Zemlya (*n* = 13) and mainland Russia (Taimyr; *n* = 12), assuming Novaya Zemlya as the population most closely related to the Svalbard reindeer (Dussex et al. 2023). We used as input the BAM files generated with GenErode and downsampled them to the depth of the genome with the lowest coverage (ie 4×). We then calculated the PBS in ANGSD v0.917 (Korneliussen et al. 2014) which picks one allele randomly. We calculated genotype likelihoods and the sample allele frequency (saf) with the following arguments: -doSaf 1; -gl 1; -minMapQ 30; -minQ 30. To generate the site frequency spectrum (SFS), we generated an ancestral genome based on red deer (*Cervus elaphus*; ERR6054834-ERR6054835), moose (*Alces alces*; ERR4659222), and mule deer (*O. hemionus*; SRR6433393) to polarize our multi-individual vcf (Heckeberg 2020). We mapped those reads to the caribou assembly as described above, subsampled each of these three genomes to the minimum depth of coverage (ie 10×) and merged them with SAMtools *merge*. We then used ANGSD to generate the consensus ancestral genome using the doFasta 2 and doCounts 1 options. We then used this ancestral genome for -anc argument to estimate the unfolded SFS. Next, we used the *realSFS* command to calculate the 2D SFS for each pair of populations with the following options: -P 10; -r. We then used the *realSFS fst index* command to generate *F*_ST_ binary files and extracted *F*_ST_ values with the following options: -win 50,000; -step 10,000. Finally, we extracted the PBS values for outlier windows overlapping with the 99.9th percentile of the distribution in R (R Core Team 2020) and considered these candidates for positive selection. We then used BEDTools v2.29.2 *intersect* (Quinlan and Hall 2010) to find the overlaps between these outlier windows and coding regions in our annotation.

In order to test whether outlier regions were the result of positive selection or genetic drift associated with the recent population decline, we used ANGSD to calculate the neutrality test statistic Tajima's D (Tajima 1989) in all three reindeer populations. For this, we employed the *realSFS* function to estimate SFS and neutrality indices with the *thetaStat do_stat* the following arguments: -win 50,000; -step 10,000.

### Loss-of-Function Alleles

Based on the hard call genotypes from GenErode, we used SnpEff v4.3 (Cingolani et al. 2012) to annotate synonymous and nonsynonymous variants in coding regions. We removed gene models with in-frame STOP codons, missing START and terminal STOP codons from our annotation using the “-J” option and genes labeled as pseudogenes using the “--no-pseudo” option in Cufflinks. In SnpEff, we identified two categories of variants: (i) moderate impact, nondisruptive variants that might change protein effectiveness (ie missense), and (ii) high impact, variants assumed to have high (disruptive) impact on protein, probably causing protein truncation, loss of function, or triggering nonsense-mediated decay (eg stop gained codons, splice donor variant and splice acceptor, and start codon lost). We also excluded intergenic variants and introns using the “-no-intergenic” and “-no-intron” options.

We then estimated allele frequencies in genomes from Svalbard (*n* = 28) and Russia (*n* = 25; Novaya Zemlya and Taimyr; Table S1) by only retaining sites scored in at least half of the genomes in each population. To select sites under putative selection in Svalbard genomes, we considered alleles with a frequency ≥0.7 in Svalbard and ≤0.25 in Russia. We used these thresholds to take into account (i) the effect of drift associated with overhunting, which could have counteracted the effect of selection and reduced the frequency of some adaptive alleles and (ii) because selection is more likely to have acted on standing variation after the recent colonization of Svalbard instead of on de novo mutations.

### Copy Number Variation (CNV)

Based on the hard call genotypes from GenErode, we then used CNVcaller (Wang et al. 2017) to identify copy number variants in Svalbard reindeer. We first split the chromosomes of the caribou assembly (GCA_019903745.2) using *seqtk* (https://github.com/lh3/seqtk) and then split each chromosome into short k-mer sequences using the *0.1.Kmer_Generate.py* script from CNVcaller with a window size of 1,000 bp. The k-mer fasta file was then aligned to the assembly by first creating a fatsa.sa file with *blasr* v5.3.2(Chaisson and Tesler 2012) *sawriter* followed by k-mer alignment with *bastr* using the *-m 5 --noSplitSubreads --minMatch 15 --maxMatch 20 --advanceHalf --advanceExactMatches 10 --fastMaxInterval --fastSDP --aggressiveIntervalCut --bestn 10* options. The *0.2.Kmer_Link.py* script from CNVcaller was then used to generate duplicate window records files for the 34 autosomal chromosomes and the X chromosome and subsequently combined into a single link file for all chromosomes. The reference genome was segmented into 1,000 bp overlapping sliding windows using the *CNVReferenceDB.pl* script from CNVcaller with a lower GC content limit of 0.2, an upper GC content limit of 0.7, and an upper gap limit of 0.5. The bam files for each individual were filtered using samtools v.1.18 to exclude PCR duplicates (flag: 0 × 400), nonprimary alignments (flag: 0 × 100), supplementary alignments (flag: 0 × 800), and reads with a mapping quality below 30. The read depth for each individual and window was then analyzed using the *Individual.Process.sh* script from CNVcaller, specifying “CM056435.1” (X chromosome) as the sex chromosome. The normalized read depth files of the 53 individuals were used for CNV discovery with the *CNV.Discovery.sh* script from CNVcaller with a minimum of Pearson's correlation coefficient between two adjacent nonoverlapping windows (-r) of 0.3 as recommended for sample sizes between 50 and 100, three homozygous gain/loss individuals for candidate CNV window definition (-h), and a minimum frequency of 0.1 for gain/loss individuals, followed by CNV genotyping for each individual using the *Genotype.py* script from CNVcaller.

To identify CNVs that are highly differentiated between Svalbard and Russia, we used the *dapc()* function from the adegenet package in R v4.3.1 to perform a Discriminant analysis of principal components (DAPC). The first five principal components and the first discriminant (as only two groups were compared) were used for the analysis. Loadings for each variant were estimated with the *loadingplot()* function, and a threshold of 0.0005 was used to identify highly differentiated CNVs. To identify genes where coding regions (CDS) overlap with these highly differentiated CNVs, the *subsetByOverlaps()* function from the GenomicRanges R package was used with the genome annotation file.

We then estimated CNV allele frequencies for Svalbard (*n* = 28) and Russia (*n* = 25; Novaya Zemlya and Taimyr; Table S1). We considered deletions (ie coded as CN0) and duplications (ie coded as CN2, CNH) and also the ancestral allele (ie coded as A; unique gene copy). For duplications, we did not discriminate between two (CN2) or multiple duplications (CNH).

### Large Deletions

We also identified large deletions (>500 bp) fixed in Svalbard reindeer relative to Russian and Norwegian reindeer using the approach described in van der Valk et al. (2022) (https://github.com/tvandervalk/aDNA-deletions) and the data mapped to caribou (GCA_019903745.2). This approach is based on the comparison of the genome depth of coverage of a focal species and an outgroup. Genomic regions without any read mapping in the focal species but with normal read depth of coverage for the outgroup represent deletions specific to the focal species. We analyzed the bam files for Svalbard reindeer genomes (*n* = 28) and genomes from an outgroup (ie Norway and Russia; *n* = 11; Table S1) from different localities used in the previous analyses and with depth ≥10× and (Table S1). The method first creates a mappability map by fragmenting the assembly of the caribou reference genome into overlapping, 50 bp reads, by sliding across the reference sequence in overlapping windows of 50 bp using a step size of 1 bp and recording the sequence for each window. We then mapped these reads back to the reference using the same parameters as described above and filtered out all nonuniquely mapping reads. Next, we used a strict coverage threshold, recording all sites with depth below 25 as unmappable into a bed file. All unmappable regions within 250 bp from each other were then merged into one larger region using BEDTools *merge* command (-d 250) (Quinlan and Hall 2010).

Next, we estimated the depth per site for each resequenced genome with SAMtools *depth* (Li et al. 2009) and filtered reads below mapping quality of 30 (-Q 30) and shorter than 50 bp (-l 50). The total depth per site was then calculated as the sum of the total depth at a site for all reindeer genomes. Summing the depth over several genomes from the same species should give a value of zero for fixed deletions but will increase the depth of surrounding regions by the factor of genomes (assuming equal depth among the genomes). This results in increased power for distinguishing deletions from surrounding regions with average coverage. Summing the depth per population, excluding unmappable regions, resulted in an average total coverage per site of 644 and 123 for the merged Svalbard reindeer and Norwegian/Russian genomes, respectively.

Next, we used a sliding window approach to identify regions without sequence read support (eg regions without read coverage) in the Svalbard reindeer genomes but with good coverage support in Norwegian/Russian genomes. Identified windows that were adjacent to each other were then merged using BEDTools *merge*. Deletions broken due to unmappable regions were also merged. On rare occasions, a deleted region contains aligned reads either due to misalignments or the mismapping of nonendogenous DNA sequences. To account for such effects, we additionally merged deletions into a single large deletion if they were within 250 bp from each other and the region separating the two deletions had a coverage lower than 5% of the expected Svalbard reindeer coverage (ie <7.7). We restricted this analysis to the 34 autosomes. Finally, we used the BEDTools *intersect* to find the deletions in the resulting bed file overlapping with coding regions from our annotation.

### Gene Ontology

For all 120 genes identified in the PBS, SNPeff, CNV, and large deletion analyses, we extracted candidate genes and used GOrilla (https://cbl-gorilla.cs.technion.ac.il/) to perform a gene ontology enrichment analysis and Panther (http://pantherdb.org/; Mi et al. 2010) using mouse as reference dataset to identify the biological functions of candidate genes identified. We also used the Mouse Genome Informatics database (MGI; www.informatics.jax.org; Blake et al. 2014) as well as literature searches to manually retrieve gene ontologies and mammalian phenotype information for each candidate gene. The complete set of 31,659 annotated Svalbard reindeer genes was used as the reference set, and the 120 genes identified with PBS, SNPeff, CNV, and large deletion analyses were used as the test set.

## Supplementary Material

evaf160_Supplementary_Data

## Data Availability

The genomic resequencing data newly generated for this study are available under the European Nucleotide Archive (ENA) accession code PRJEB80888. Other resequencing data utilized in the study are available under ENA accession codes PRJEB57293, PRJEB61721, and PRJEB37216. Code for data processing and analysis and simulation are deposited to GitHub: https://github.com/ndussex/Reindeer_adaptation.
