# Supplementary material for: The Genomic Basis of the Svalbard Reindeer's Adaptation to an Extreme Arctic Environment
Source: Genome Biol Evol. 2025 Aug 13;17(9):evaf160. doi: 10.1093/gbe/evaf160 (PMC12409278; doi:10.1093/gbe/evaf160)
Supplement: evaf160_Supplementary_Data [file evaf160_supplementary_data.zip › Supplementary material - Dussex et al..docx]

**Supplementary material**


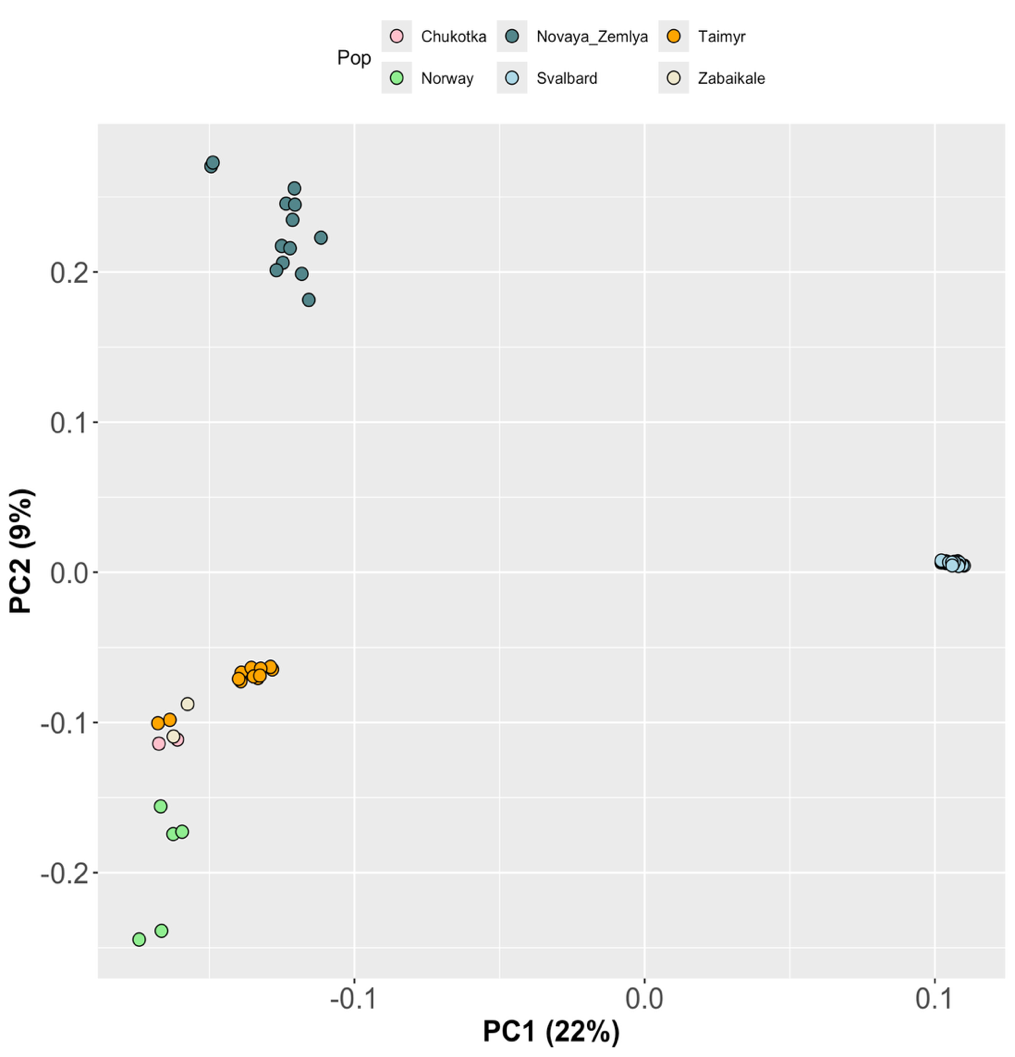

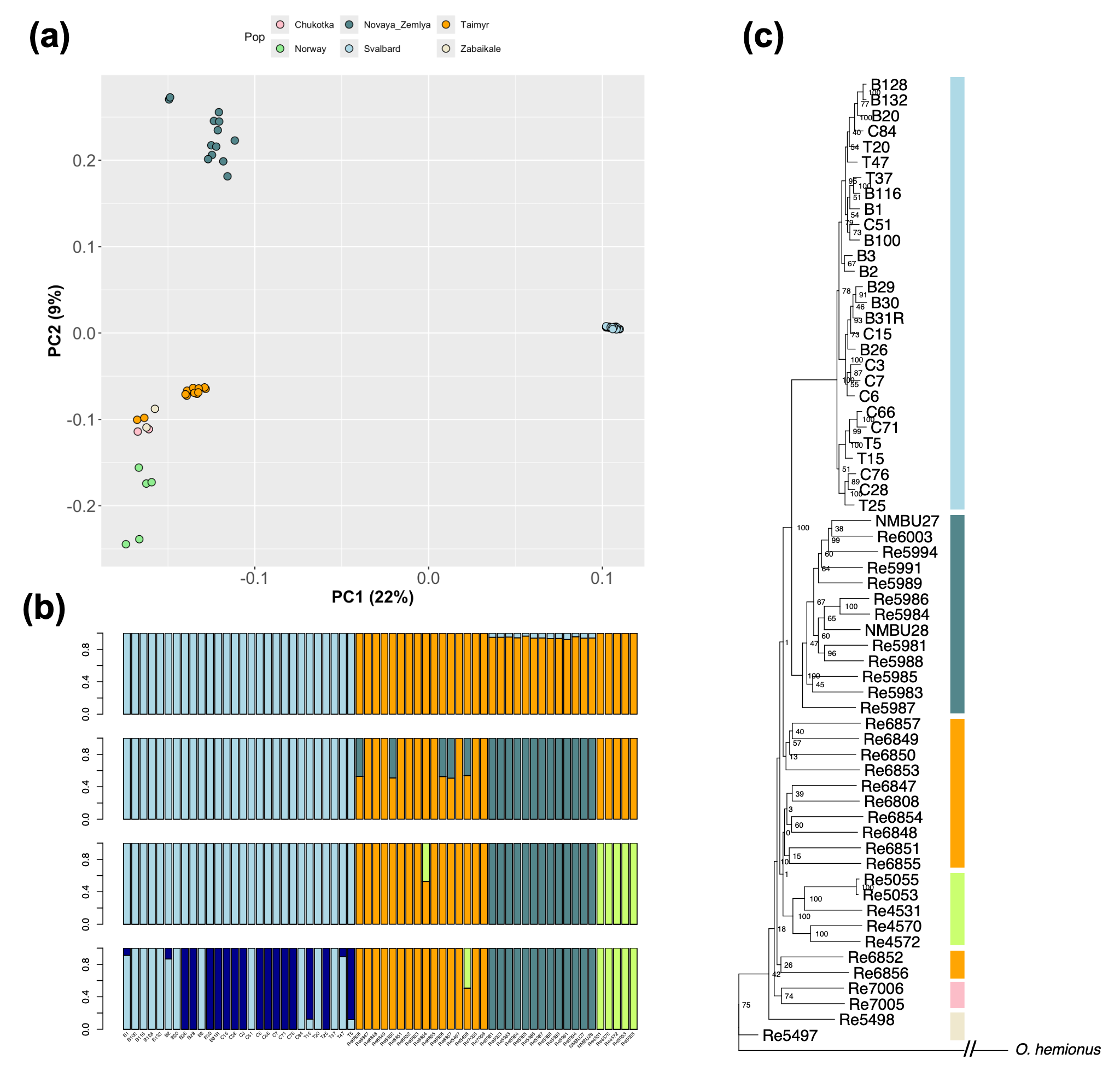


**Figure S1.** Maximum Likelihood phylogeny using *Odocoileus hemionus* as outgroup with bootstrap values obtained from 100 bootstrap replicates.


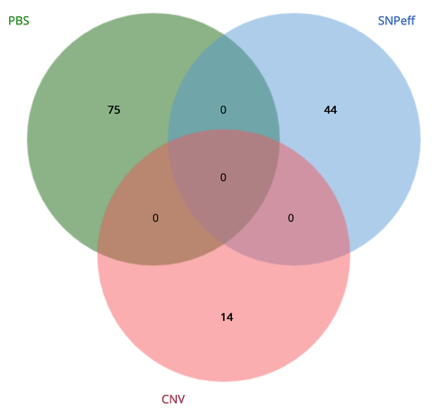


**Figure S2.** Venn diagram showing the overlap for coding regions identified under putative selection among the three approaches used (i.e., population branch statistic, PBS; SNPeff; copy number variation, CNV)
